# Advanced Hybrid System for Ammonium Valorization as Liquid Fertilizer from Treated Urban Wastewaters: Validation of Natural Zeolites Pretreatment and Liquid-Liquid Membrane Contactors at Pilot Plant Scale

**DOI:** 10.3390/membranes13060580

**Published:** 2023-06-02

**Authors:** Álvaro Mayor, Mònica Reig, Xanel Vecino, José Luis Cortina, César Valderrama

**Affiliations:** 1 CETaqua, Carretera d’Esplugues, 75, 08940 Cornellà de Llobregat, Spain; amayor@cetaqua.com (Á.M.); jose.luis.cortina@upc.edu (J.L.C.); 2 Chemical Engineering Department, Escola d’Enginyeria de Barcelona Est (EEBE) Universitat Politècnica de Catalunya (UPC)-BarcelonaTECH, C/Eduard Maristany 10-14, Campus Diagonal-Besòs, 08930 Barcelona, Spain; cesar.alberto.valderrama@upc.edu; 3 Barcelona Research Center for Multiscale Science and Engineering, Campus Diagonal-Besòs, 08930 Barcelona, Spain; 4 CINTECX, Chemical Engineering Department, Campus as Lagoas-Marcosende, University of Vigo, 36310 Vigo, Spain; xanel.vecino@uvigo.es

**Keywords:** nitrogen, recovery, ion exchange, scale up, demonstration, circular economy

## Abstract

This study evaluates a hybrid system combining zeolites as a sorption stage and a hollow fiber membrane contactor (HFMC) for ammonia (NH_3_) recovery from treated urban wastewater. Ion exchange with zeolites was selected as an advanced pretreatment and concentration step before the HFMC. The system was tested with wastewater treatment plant (WWTP) effluent (mainstream, 50 mg N-NH_4_/L) and anaerobic digestion centrates (sidestream, 600–800 mg N-NH_4_/L) from another WWTP. Natural zeolite, primarily clinoptilolite, demonstrated effective desorption of retained ammonium using a 2% NaOH solution in a closed-loop configuration, resulting in an ammonia-rich brine that enabled over 95% NH_3_ recovery using polypropylene HFMCs. A 1 m^3^/h demonstration plant processed both urban wastewaters, which were pretreated by ultrafiltration, removing over 90% of suspended solids and 60–65% of COD. The 2% NaOH regeneration brines (2.4–5.6 g N-NH_4_/L) were treated in a closed-loop HFMC pilot system, producing 10–15% N streams with potential use as liquid fertilizers. The resulting ammonium nitrate was free of heavy metals and organic micropollutants, making it suitable for use as liquid fertilizer. This comprehensive N management solution for urban wastewater applications can contribute to local economies while achieving reduced N discharge and circularity goals.

## 1. Introduction

United Nations projections predict that the global population will reach 8.6 billion by 2030 and 9.8 billion by 2050 [1]. This growth in population and consumption will put immense pressure on the food industry to increase production, requiring more intensive agricultural practices and higher usage of land, water, energy, and fertilizers [2]. Non-renewable mineral fertilizers, such as N, P, K, and Mg, currently form the basis of modern agriculture. However, crops only absorb 31–49% of supplied N and 35% of supplied P.

Wastewater treatment has long been seen as a health and environmental concern. Traditional wastewater treatment technologies, established in the early 20th century, were designed to remove organic pollutants (OMPs) and nutrients like nitrogen and phosphorus, but are not considered sustainable [3]. Recently, the perspective on wastewater has shifted, transforming from a health concern to a valuable source of nutrients for fertilization. This change has been driven by phosphorus scarcity and the environmental impact of nitrogen-based fertilizer production [4].

As a result, research has been focused on developing new treatments and procedures to turn wastewater treatment plants from energy consumers into energy recovery and nutrient production facilities [5]. Various alternative technologies have been explored for ammonium removal from urban and industrial wastewater, including ion exchange, adsorption, biological technologies, air stripping, chemical precipitation, chemical oxidation, and membrane-based technologies. Each method has its advantages and limitations, including cost, removal rate, sensitivity to pH and temperature, and the introduction of new pollutants [6]. However, when promoting nutrient circularity within urban and industrial water cycles, only certain technologies, or combinations of technologies (e.g., hybrid processes) are deemed suitable.

Ion exchange using low-cost sorbents, i.e., natural [7] and synthetic [8] zeolites, appear to be a promising option due to the capability to recover ammonium from treated wastewaters after an ion exchange reaction after the exchange of the NH_4_^+^ in solution by the counter ion (e.g., typically Na^+^) of the zeolite. The first option is the recovery from the main streams of the conventional activated sludge (CAS) systems or the main streams after a treatment with An-MBR (anaerobic membrane bioreactor) when residual values of NH_4_^+^ may range between 5 to 60 mg/L. A second option of ammonium recovery is centered in its recovery from the sidestreams generated in the anaerobic digestion of sewage sludge, where ammonium concentrations between 600–900 mg/L NH_4_^+^ are expected to be treated [9]. Once the zeolites are saturated with NH_4_^+^ ions, zeolites can be regenerated with NaCl, KCl, NaOH or mixtures of them and then a second stage is needed to recover the N-species (NH_3_) present in regeneration brines as a valuable pure by-product (e.g., ammonium salts as fertilizers [10] or ammonia [11]). It has been also reported a wide variety of sorption capacity towards ammonium, ranging from 2–43.5 mg N-NH_4_/L depending on the zeolite properties and the composition of the treated stream (e.g., ammonium concentration and concentration of competing ions) [6,12]. Besides, the cost of zeolites is much lower than the cost of synthetic zeolites or polymeric sorbents, which provides the potential to be implemented at full scale facilities [6,12]. However, the application of zeolites at full scale still must face and overcome several challenges. One of the most important is the regeneration stage and the chemicals used (i.e., NaOH, NaCl or KCl) required to maximize nitrogen recovery. To achieve recovery efficiencies up to 90–98% doses of NaCl up to 50–80 g/L may be required, which would mean 50–60% of the operational expenditure of the process [13]. Other main issue to be addressed is the presence of competitive ions such as potassium, calcium, or magnesium, which are also adsorbed by the zeolites, decreasing the exchange capacity towards ammonium. This effect will be increased treating anaerobic digestion centrates due to concentrations of K, Ca and Mg may reach, on average, values of 350 mg/L, 300 mg/L and 90 mg/L_,_ respectively [14]. Moreover, the lifespan of the zeolites is still one of the key parameters to define the operating cost of this technology and it is also one of the parameters scarcely evaluated in literature. It has been reported that zeolites can be regenerated 12 times with 0.6 M NaCl at pH 10 [15] without losing capacity but it has been also reported a loss of zeolite mass between 2.4–5.7%weight after 10 cycles of regeneration due to caustic attrition [16].

Once the zeolites are regenerated a N-rich solution is produced. This solution must be treated to recover and valorize the ammonium. This way liquid-liquid membrane contactors (LLMC) appear as a promising technology to achieve this objective.

The LLMC is a novel and ecofriendly technique where two liquid phases are separated by a membrane and target specie is only transported by diffusion phenomena, due to phases do not mix between them [17]. For that, this technique allows ammonia recovery from wastewater effluents using an acid as stripping solution in several industrial applications [18]. Then, its transformation into ammonium salts is produced and they could be used as liquid fertilizers such as NH_4_NO_3_, (NH_4_)_2_SO_4_, (NH_4_)_2_HPO_4_ or (NH_4_)H_2_PO_4_, among others [19,20,21].

Licon Bernal et al. [19] and Sancho et al. [20] studied the applicability of hollow-fiber membrane contactors for the recovery of ammonium from a synthetic regenerant solution, which was used as a surrogate for the liquid phase produced during the regeneration of loaded zeolites being applied in tertiary wastewater treatment. Results showed 95% ammonium recovery; therefore, the treated regenerant solution can be reused for zeolite regeneration reducing the operating costs. Garcia-González & Vanotti investigated the nitrogen recovery from swine manure using hollow-fiber membranes directly submerged into the ammoniacal manure. The investigation of the influence of pH adjustment and aeration rate of swine manure at different ammonium concentrations resulted in ammonium recovery rates up to 94% [22].

Investigations with membrane contactor at pilot-scale have been conducted at Neugut WWTP (Switzerland). Process water from sludge dewatering deriving from different municipal WWTPs and a digester with codigestion of meat waste processing and containing 700–3400 mg/L NH_4_-N was treated by a pilot plant including three membrane stages in series with 120 m^2^ total membrane surface area [21]. Sulphuric acid was recirculated through the lumen side of the hydrophobic hollow-fiber membranes [18]. Boehler et al. achieved elimination rates between 80% and 99% depending on the adjustment of pH value and temperature (up to pH 10.5 and 54 °C using caustic soda and heat exchange, respectively). A full-scale membrane contactor facility was implemented at Yverdon les Bains WWTP (Switzerland). The facility includes several pretreatment steps (alkali addition, heat exchanger and multiple filtration steps) and membrane modules in series. First operational experiences revealed about 70% nitrogen elimination when the pH value and temperature of the process water were adjusted to pH 9.7 and 40 °C, respectively [23].

The primary objective of this study is to evaluate the technical feasibility of utilizing natural zeolites and LLMCs (liquid-liquid membrane contactors) for recovering and repurposing ammonium from wastewater treatment plants (WWTPs) as a fertilizer. Zeolites were examined as an advanced pretreatment and concentration method prior to implementing LLMCs. Two separate streams were tested: WWTP effluent (diluted stream containing 50 mg N-NH_4_/L) and anaerobic digestion centrates (concentrated stream containing 600–800 mg N-NH_4_/L).

Initially, a laboratory-scale study was conducted to determine the optimal operating conditions using effluent mainstream wastewater from the Vilanova i la Geltrú (VNG) WWTP and a natural zeolite (named ZEOCAT) with a particle size of 1.0–2.5 mm. Subsequently, these conditions were tested at a zeolite pilot plant situated at the VNG-WWTP, operating for one year. Following this, the pilot plant was transported to Murcia ESTE WWTP to operate with anaerobic digestion centrates for another year.

Upon evaluating the zeolites’ superior performance in terms of cation capacity and concentration factor, the decision was made to concentrate the valorization efforts within the sidestream, as the lower flow rates and higher nitrogen concentrations would yield better economic performance. The operation of the LLMCs was aimed at producing a high-quality ammonium salt suitable for use as fertilizer, ensuring it is free of heavy metals and organic micropollutants.

## 2. Materials and Methods

### 2.1. Water Characterization

Wastewater used at lab experiments was sampled from the effluent of VNG-WWTP. It was collected from the effluent of the secondary clarifier. On the other hand, the sidestream used at pilot-scale was collected from Murcia ESTE WWTP. The composition of each sample was characterized measuring pH, conductivity, TSS and dissolved ions. pH and conductivity were measured with Crison pH meter GLP 22 and Crison EC-Meter GLP 31, respectively. Total suspended solids were measured according to the procedures described in the Standard Methods for the Examination of Water and Wastewater [24]. Analysis of cations such as Na^+^, NH_4_^+^, K^+^, Mg^2+^ and Ca^2+^ was carried out by means of cationic chromatograph Thermo Fisher Dionex ICS-1000. Analysis of anions such as anions Cl^−^, NO_3_^−^, PO_4_^3−^ and SO_4_^2−^ was carried out by means of a chromatograph Thermo Fisher Dionex ICS-1100.

### 2.2. Characterization of Zeolites

Natural clinoptilolite zeolites were provided by ZEOCEM (www.zeocem.com), with two different granulometries: from 0.5–1.0 mm to 1.0–2.5 mm of mean particle diameter. Morphology and composition of the raw sample and samples after being operated in sorption and desorption cycles were studied by means of Field Emission Scanning Electron Microscope (FESEM) (JEOL JSM-7001F) and X-ray diffraction (XRD).

Prior to experiments, zeolites were washed with distilled water to remove dust and smaller particles. After that, zeolites were submerged in a 1 M NaOH and stirred in an orbital stirrer for 1 h. This process allows to activate the zeolite into its Na^+^ form, and thus increasing its ammonium exchange capacity. Finally, zeolites were washed out with distilled water to adjust pH to neutral values (7–8) and loaded in the fixed-bed columns.

### 2.3. Column Laboratory Experimental Setup

The lab-scale experimental setup consisted of two fixed-bed columns with 18.7 cm high and 22.6 mm internal diameter, each column had glass wool at the bottom to prevent zeolites from running off. Furthermore, inert sand was added until filling 10% of the column to promote flow distribution. Finally, column was loaded with 67 g of zeolite.

During the adsorption phase, feed stream entered from the bottom of the column and left the column from the top. When the zeolites were exhausted and a regeneration was required, a first step of counter current (from top to bottom) washing at 4 times feed flow was developed to expand the zeolite bed. After that, the regeneration took place from bottom to top (co-current). Finally, once the zeolite was regenerated a last step with Milli-Q water in the same flow conditions as adsorption was carried out to adapt the zeolite bed.

To determine the porosity of the zeolites column beds once packed, a 10 mL lab cylinder was tared and a certain number of zeolites ($W_{0}$) were poured into and the occupied volume was noted ($V_{zeolite}$). Distilled water was then added until the 10 mL level mark. It was then stirred for 1 h in an orbital shaker to compact the zeolites. Finally, excess water was removed until the level matched the level of zeolites, then the cylinder was weighted again ($W_{1}$), the difference between both is the weight of water ($W_{water}$) and thus the volume of water ($V_{water}$) was estimated. The porosity of the beds was estimated using Equations (1) and (2):

(1) $$W_{0}-W_{1}=W_{water}\approx V_{water}$$

(2) $$\frac{V_{water}}{V_{zeolite}}\cdot 100=porosity$$

Porosity of the columns used in the column experiments were 0.42 for experiments with 2.5 mm zeolite and 0.39 for 0.1 mm zeolite mean diameter, respectively.

#### Experimental Design

Mainstream water was used for this first set of experiments at lab-scale. In this case, to maximize the nitrogen recovered, different flow rates at the sorption and regeneration steps were evaluated. A total of four experiments, each of which was carried in duplicate, tested different couples of sorption and regeneration flow rates. Each experiment consisted in an adsorption and a regeneration step and between both phases Milli-Q water was circulated counter current for 10 min to rinse the column. The experimental conditions for each of the experiments are summarized in Table 1.

To achieve optimal regeneration, 11 regenerant solutions were tested al lab-scale, hence a certain amount of zeolite was loaded and saturated using the fixed-bed configuration set-up.

Thus, to study the regeneration process 67 g of zeolite were loaded in a column of 18.7 cm high and 22.6 mm internal diameter. After that, zeolites were fed with VNG-WWTP effluent until saturation was achieved. It was considered that the breakthrough point was reached when the concentration of N-NH_4_ measured at the effluent was higher than 20% of N-NH_4_ at the influent (C/C_0_ = 0.2). For an influent with 50 mg/L N-NH_4_, C/C_0_ = 0.2 matched 10 mg/L of N-NH_4_ in the effluent which was the threshold established by WW Directive (91/271/EEC) for sensitive zones. From the operating point of view, breakthrough point may be achieved upon reaching C/C_0_ = 1 which is the saturation point. The total mass of loaded zeolite was split into 11 fractions of 5.5 g and put into conical flasks, in which 55 mL of regenerant solution was poured (maintaining a ratio of 1:10 g_zeolite_/mL_regenerant_). Regenerant solutions were based on NaOH (0.05; 0.1 and 0.5 mol/L), NaCl (1; 5; 10 and 20 g/L) or a mixture of both substances (0.01 mol NaOH/L and 1 g NaCl L; 0.01 mol NaOH/L and 20 g NaCl/L; 0.1 mol NaOH/L and 1 g NaCl/L; 0.1 mol NaOH/L and 20 g NaCl). These conical flasks were shaken for 24 h, and the supernatant was finally collected and analyzed by ion chromatography.

### 2.4. Zeolites Pilot Column Experimental Setup

**Figure 1:**
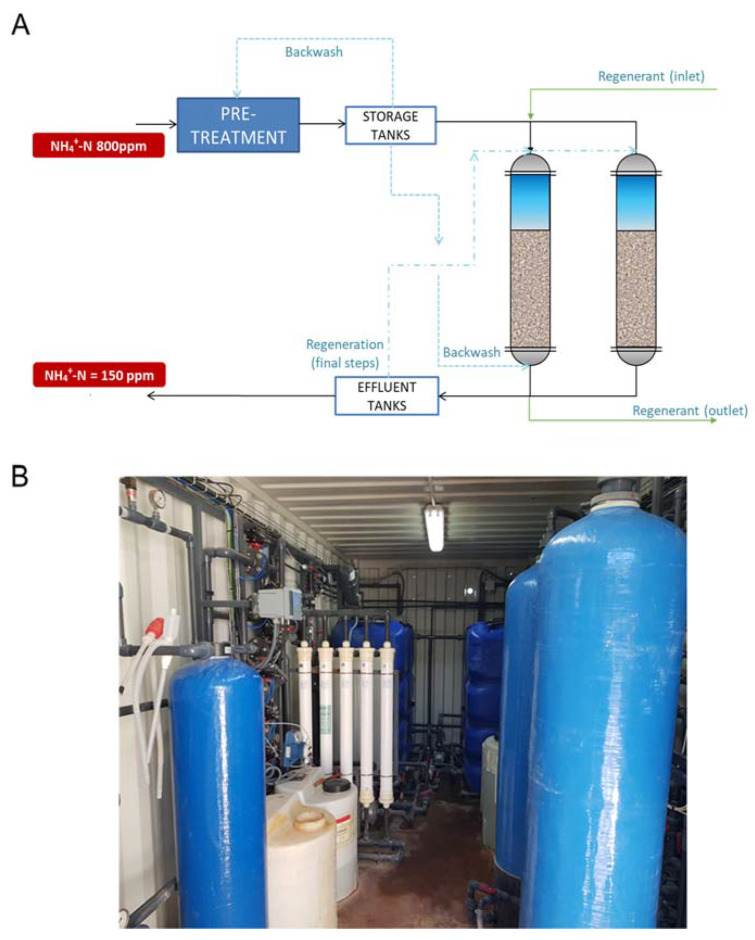
(**A**) Sschematic flow diagram of the pilot plant; (**B**) Photograph of the pilot plant.

Considering the results achieved at lab-scale, a pilot plant with 1 m^3^/h of nominal capacity was designed, built, and installed in VNG-WWTP to operate for a year and then moved to Murcia ESTE WWTP to operate another year. Feed water for this pilot plant consisted of VNG-WWTP effluent as mainstream water and Murcia Este Centrates, as sidestream water. The feed stream was pre-treated by a filtration system based on a glass filter (0.5–1.0 mm) and an ultrafiltration unit with 50–60 kDa size cut-off working in dead-end configuration. Ultrafiltered water was then fed to the zeolite fixed-bed columns as can be seen in Figure 1.

Two columns, each with a capacity of 200 L, were loaded with 100 kg of zeolites provided by ZEOCEM, with two different particle sizes (0.5–1.0 mm and 1.0–2.5 mm). The purpose was to test the effect of particle size on capacity, considering that smaller particles have higher capacity but may also cause operational issues. These columns were designed to offer operational flexibility, allowing them to be operated in series, parallel, or with just one column using electro-valves.

A 150 L high-density polyethylene tank was used to collect and continuously feed the pre-treatment system with the feed flow. The permeate stream from the UF unit was stored in two high-density polyethylene tanks with a total volume of 1500 L and then pumped to the zeolite columns. Ammonium-free water was stored in two high-density polyethylene tanks with a total volume of 1500 L for later use in preparing the regenerant solution. All reservoir tanks were covered and vented to maintain atmospheric pressure. A process logic controller (Simatic S7-1200 controller, SIEMENS) was employed to control, operate, and log operational data from the pilot plant.

The zeolites pilot plant operated continuously for one year, testing each different stream. Each experiment involved sorption until the breakthrough point was reached, followed by zeolite regeneration. The sorption tests focused on evaluating the cation exchange capacity (CEC) for N-NH_4_ while maintaining a low concentration of N-NH_4_ (<1 ppm) in the effluent. Once the zeolites reached the breakthrough or saturation point, they were regenerated to recover their ion exchange capacity.

#### Experimental Design in Mainstream and Sidestream

This study evaluated the performance of zeolite in recovering ammonium from mainstream and sidestream processes of WWTPs. We compared the adsorption efficiency and capacity of zeolites under different operating conditions.

Four mainstream and four sidestream adsorption experiments were conducted, maintaining constant operating conditions to assess process stability and measure CEC decrease over cycles.

For mainstream experiments, a single column (100 kg zeolites) was used to save time. Zeolites from ZEOCAT (particle size: 1.0–2.5 mm) were fed at a flow rate of 400 ± 50 L/h using a centrifuge pump (MS 100/0.55).

Regeneration tests aimed to maximize ammonium recovery and used NaOH 0.5 M regenerant solution for high sodium concentration and pH. Ammonium levels were measured in the influent, effluent, and during regeneration to assess nitrogen mass balances.

The regenerant solution was not sampled during regeneration but stored as a homogeneous sample for later analysis.

In sidestream conditions, zeolite columns were fed at a flow rate of 400 ± 50 L/h using ZEOCAT zeolites (particle size: 0.5–1.0 mm). Column regeneration used NaOH 0.2 M solution (350–500 L). The regenerated stream was stored in a 1 m^3^ container for subsequent membrane contactor experiments. A summary of the experimental design is displayed in Table 2.

### 2.5. Liquid-Liquid Membrane Contactors Pilot Experimental Setup

**Figure 2:**
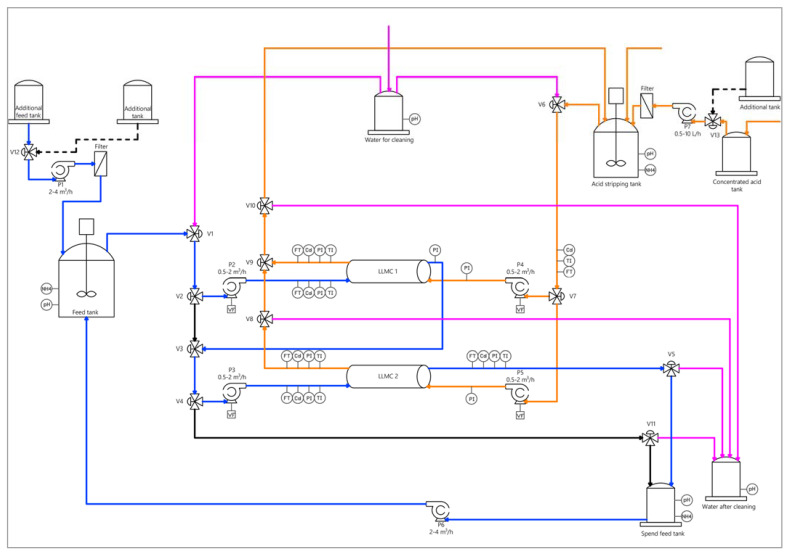
Scheme of the LLMC pilot plant.

LLMC unit separates two liquid phases by hydrophobic porous hollow fiber membrane (3M). The LLMC system for ammonia recovery, working in a closed-loop configuration, can be described as follows (Figure 2): ammonia-rich wastewater is fed on the feed side (lumen side) while the acid stripping solution is fed on the on the opposite side (shell side). Then, the ammonia recovery process inside the LLMC takes place: ammonia gas diffuses from the feed stream to the feed-membrane interface, volatilizes inside it and then diffuses through the membrane pore of the membrane to reacts with the acid stripping solution on the shell side. In this sense, hydrophobic porous membranes provide a safe technology to assure ammonia recovery from WWTP since the potential organic and inorganic micropollutants transport on the generated by-product is avoided [19,25].

The possibility of using different acids allows to produce fertilizers on demand and consequently to increase their potential market penetration. In this case, using HNO_3_ (0.4 M), as the acid in the stripping solution, produces a nitrogen fertilizer (which is a single-nutrient fertilizer); whereas when H_3_PO_4_ is used as a stripping solution, a nitrogen-phosphorous fertilizer (which is a multi-nutrient fertilizer) is obtained. There is a control loop that can maintain the pH at a desired value adding the acid HNO_3_ (58%) through the time. In this case, the set point was stablished to keep the pH at 3.4 and maximize the mass transfer. On the other hand, an exhausted effluent, free of ammonia is also obtained during the LLMC process. This stream, at high pH, can be reused for the zeolite’s regeneration [11,26].

Since water transport is an unwanted process associated with ammonia transport due to differences in vapor pressure between the feed side and the acid side, drying was performed after conducting an experiment.

The drying time was around 8 h at 40 °C with a flow rate of 34 m^3^/h. The maximum gas pressure was 0.70 kg/cm^2^. For this reason, the gas line had pressure, temperature, and flow sensors.

Table S1 can be found in supplementary material and shows the main parameters considered for the design and operation of the unit.

#### Experimental Design

Prior to testing the LLMCs, two fully characterized experiments were conducted to validate their performance. These experiments provided a solid foundation for understanding the capabilities and limitations of the system, enabling further optimization of the process.

All the experiments described in this part were carried out with regenerant solution from zeolites treating sidestream, which produced a regenerant stream with 4–5 g/L N-NH_4_. However, the first one only has 1.3 g/L N-NH_4_ due to it was stored at pH 12 for a long time before the experiment which led to N loses as ammonia gas.

Following the successful completion of these initial experiments, the system operated in batch mode (producing a total of 9 lots of fertilizer), allowing for analysis only at the beginning and end of each experiment. N-NH_4_ concentration was measured using kits during the experiments on the feed side to determine the end of the operation. Kits cannot be used in the acid side due to acidic conditions and high concentrations, requiring distillation and titration. Each experiment started with 10 L of HNO_3_ 0.4 M in the acid side to start the operation, after that, the control loop added HNO_3_ (58%) to keep the pH at 3.4 and maximize the mass transfer. Full characterizations (N-NH_4_, NO_3_, ions, heavy metals, and OMPs) were conducted for each batch of fertilizer produced. After each experiment, the membrane underwent a drying procedure to remove any potential water in the pores, preventing pore wetting in subsequent experiments.

Additionally, due to the scale factor, 2 or 3 zeolite experiments were needed to produce 1 m^3^ of N-rich stream required to start the LLMC tests. On the other hand, one of the most critical factors to monitor and avoid is pore wetting. Although the membranes are hydrophobic, pores may become wet during operation. This issue depends on the pressure and the selected acid. If too many pores become wet, constant water transport through the membrane occurs, leading to quality issues in the final product.

The summary of the experiments developed with the LLMCs are displayed in Table 3.

### 2.6. Sampling and Analytical Methods

The pilot plant was equipped with two real-time ammonium sensors (AN-ISE SC, Hach Lange)—one at the inlet of the columns and another at the outlet—to determine the ammonium uptake of the zeolites by calculating the difference between the two sensors. The PLC (programmable logic controller) was responsible for logging and controlling the measurements of both probes. When the concentration of the effluent matched that of the influent, the PLC initiated the regeneration process. Every day, two samples were taken from the influent and the effluent and sent to IRTA (Institute of Agrifood Research and Technology) for analysis. All samples were analyzed using ion chromatography (IC) to measure the concentration of soluble cations (Ca^2+^, Na^+^, K^+^, Mg^2+^) and anions (PO_4_^3−^, Cl^−^, SO_4_^2−^), as well as distillation and titration to measure N-NH_4_. Moreover, heavy metals (Al, Cr, Ni, Cu, Zn, Cd, Hg, Pb) were quantified in each ammonium-rich solution produced during column regeneration using Inductively Coupled Plasma Optical Emission Spectroscopy (ICP-OES). The content of OMPs in these ammonium-rich streams, including Celestolide, Galaxolide, Tonalide, Ibuprofen, Naproxen, Diclofenac, 4-Octylphenol, 4-Nonylphenol, Bisphenol A, and Triclosan, was analyzed using gas chromatography coupled with mass spectrometry (GC-MS). Additionally, Carbamazepine, Diazepam, Erythromycin, Fluoxetine, Roxithromycin, Sulfamethoxazole, Trimethoprim, Estrone, Estradiol, and Ethinylestradiol were analyzed using liquid chromatography coupled to mass spectrometry (HPLC-MS).

**Figure 3:**
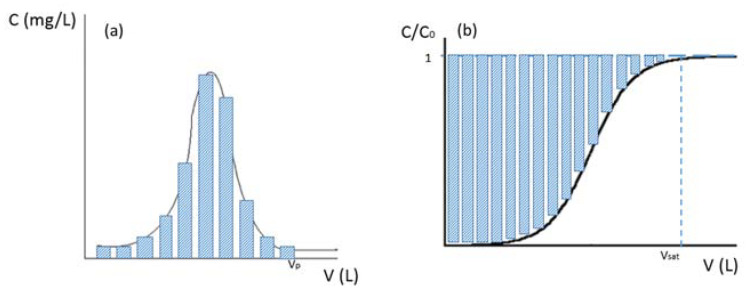
Calculations of (**a**) N uptake and (**b**) N recovery.

For the calculation of ammonium uptake, a plot (see Figure 3a) between the concentrations of the removed ions in the effluent (ordinate) versus the effluent volume (abscissa) was drawn. The breakthrough is defined as the point where the unwanted ions in the effluent appear in appreciable quantities. The extent of breakthrough increases to a point where no more ion exchange occurs, that is, the effluent contains the original concentration of the ions. This point indicates the stage at which the ion exchanger is completely exhausted and must be regenerated or replaced. For the ammonia recovered, a plot (see Figure 3b) between the concentrations in the effluent (ordinate) versus the effluent volume (abscissa) is drawn.

Other relevant parameters were such as concentration factor and % of recovery, which were calculated as follows:

The concentration factor (CF) was calculated according to the expression indicated according to Equation (3):

(3) $$CF=\frac{q_{des}\cdot \frac{m_{Z}}{V_{reg}}}{C_{0}}$$

The recovery percentage (${\%}_{recovery}$) was calculated using Equation (4):

(4) $${\%}_{recovery}=\frac{q_{des}}{CEC}\cdot 100$$

where $C_{0}$ is the initial concentration of the ion in the water (mg/L), $m_{Z}$ is the mass of zeolite in the column (g), $V_{reg}$ is the volume of regenerating solution needed in the desorption process (L), CEC is the adsorption capacity during the adsorption (g N/g_zeolite_) and $q_{des}$ is the amount of N recovered in the desorption _(_g N/g_zeolite_).

## 3. Results & Discussion

### 3.1. Water Characterization

Table 4 display the results of the wastewater characterization from mainstream and sidestream.

### 3.2. Evaluation of the Zeolites Regeneration Process at Lab-Scale

#### 3.2.1. Optimization of Zeolites Columns Operating Conditions at Lab-Scale

The adsorption results at feed flows of 4 and 10 BV/h, and its corresponding desorption results are displayed in Table 5. Considering these data, mass balances were carried out to assess the recovery effectiveness of the process, the CF and select the best combination of feed and regeneration flows.

Considering the efficiency of recovery there was no big difference from one condition to another, except for the adsorption flow of 10 BV/h and a regeneration flow of 2 BV/h which provided approximately 6% higher percentage of recovery. However, there is a direct relation between flowrate and capacity of zeolites [10,27]. As it is displayed in Table 5 selecting a feed flowrate of 10 BV/h allowed to achieve exchange capacities towards the ammonium of 9.8–9.9 mg N-NH_4_/mg of zeolites which has a direct impact in the operating costs. Thus, for the regeneration, it will always be preferred a flow 5 time higher than the feed to maximize the recovery.

The hydraulic retention time (HRT) influences the operating ammonium exchange capacity when filtering wastewater through a zeolite packed column. Beler-Baykal et al. [28] investigated the effect of the hydraulic retention time within the range 0.5–12 min. They did not recommend a hydraulic retention time of <3 min because the breakthrough would occur too fast, and they eventually selected a retention time of 5 min for their experiments. Beler-Baykal and Guven [29] found that a longer hydraulic retention time (within the interval 3–10 min) delayed the breakthrough but that most ammonium was adsorbed after 5 min. In this work, HRT from 7 to 13 min were tested. It has been assessing that in these ranges there are no significant differences since from 5 min onward 80% of the N was extracted. Considering all the experiments, it can be concluded that the mean HRT value is around 10 min which will be the desired value.

#### 3.2.2. Optimization of Regenerant Solution at Lab-Scale

When the zeolite is exhausted, the loading is interrupted, and brine is pumped through the column. Both up flow [16,30] and downflow [31] applications of the brine have been described. Many authors have used sodium chloride with the concentration 0.1–0.6 M NaCl as the regeneration. The time needed for satisfactory regeneration of the exchanger depends on the concentration and pH of the brine. Koon and Kaufmann [16] investigated the impact of pH on the regeneration performance. At pH 11.5 and 2% regeneration brine, 20 BV of regeneration brine were needed, corresponding to 1.3 h of regeneration. Koon and Kaufmann [16] found that the regeneration performance was independent of the flow rate within the interval 4–20 BV/h and chose 15 BV/h. Similar results were observed by Semmens and Porter [32], who varied the regeneration flow between 12 and 20 BV/h and continued with the regeneration flow 12 BV/h for 1 h, which is in line with the findings in this work.

When pH was increased to 12 and 12.5, 20 and 10 BV of brine were needed, respectively. In both cases, 1.2% regeneration brine was sufficient. Ødegaard [33] recommended a mixture of sodium chloride and sodium hydroxide as a regeneration brine. This would decrease the need for brine by 90% compared to using only sodium chloride. However, the advantage of using caustic regeneration brine must be put in relation to the disadvantage of possible zeolite attrition [16].

**Figure 4:**
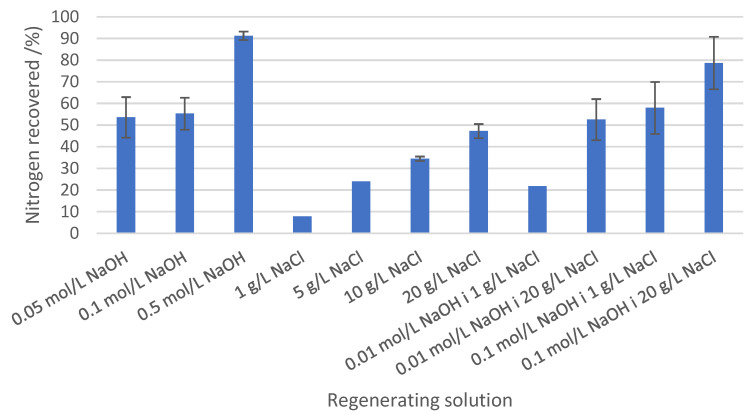
Efficiency of the regenerant solutions (%) using samples from loaded zeolites in column experiments.

The results obtained from batch desorption tests demonstrated that ammonium recovery can be achieved by providing sodium ions from NaOH, NaCl, or a combination of both. As seen in Figure 4, 90% of N recovery can be achieved with concentrations of NaOH 0.5 M, NaOH 0.1 M, and 20 g NaCl/L. Although NaOH 0.5 M provided more consistent results, possibly due to the pH increase, the optimal regenerant solution was a mixture of NaOH 0.1 M and 20 g/L of NaCl, owing to its lower cost: 3 €/kg N recovered with NaOH 0.5 M vs. 1.5 €/kg N recovered with NaOH 0.1 M and 20 g/L of NaCl

### 3.3. Zeolites Pilot Plant Performance Treating Mainstream and Sidestream

In this section, the performance of the zeolite at pilot-scale in ammonium recovery from both mainstream and sidestream processes of WWTPs is discussed. The comparison is based on the adsorption efficiency, and capacity of the zeolites under different operating conditions.

The adsorption efficiency of zeolites in mainstream processes was found to be generally lower than in sidestream processes. This is attributed to the low ammonium concentrations. In contrast, sidestream processes typically have higher ammonium concentrations which increase the cation exchange capacity.

Main results for zeolites treating mainstream and sidestream are displayed in Table 6.

As it can be seen in sidestream there are some points were the N recovered is higher than the N fed in that cycle, to understand these results is necessary to assess the overall process efficiency which is depicted in Table 7.

As observed, there is a significant impact on the CECwhen treating sidestream compared to mainstream. In sidestream treatment, it was possible to achieve CEC values of around 20 mg N-NH_4_/g, which is one of the most critical factors to consider when scaling the technology. Meanwhile, for the mainstream the highest value reached is 5.3 mg/g. Shaobin Wang and Yuelian Peng [34] reviewed different natural zeolites with different treatments and measured the CEC. They reported that the CEC could range from 2 to 30 mg N-NH_4_/g. More specifically, they reported data form a similar zeolite which is the NaOH treated zeolite with a CEC from 7.3 to 8.4 mg/g which is in the same range as the mainstream results. Being 30 mg N-NH_4_/g the highest value for a zeolite treated with microwaves, it can be considered that the value of 20 mg N-NH_4_/g achieved in the sidestream is rather high.

Another essential factor is that in this work, the lifespan of these zeolites was 4 cycles using NaOH. For sustainable operation of the process, zeolite’s exchange capacity should not be compromised during the regeneration process. It has not been found a concrete number of cycles for the lifespan of the zeolites regenerated with NaOH. However, it has been reported that the electrochemical regeneration of zeolites was investigated by Lei et al. [35]. They pointed out that the synthetic zeolites could be completely regenerated even after the regeneration solution was used for five times, and 96% of the by-product NH_3_ was converted into N_2_ [35]. An even higher regenerability was observed in the study of Huang et al. [36] where no obvious deterioration tendency was observed for NH_4_^+^ removal performance of zeolite even after 20 operational cycles.

Liberti et al. [30] investigated the performance of a pilot plant and applied a regeneration brine with the concentration 0.6 M, flow of 24 BV/h, and regeneration period of 40 min. Hlavay et al. [31] found that, if the regeneration flow rate was 5 BV/h, 4 h of regeneration was needed. However, if they increased the flow rate to 7 BV/h, the regeneration period decreased to 1.4 h. Sodium brine with the concentration 0.34 M was used.

In mainstream treatment, effluents with less than 1 mg N-NH_4_/L can be achieved, which will be crucial if discharge limits become more stringent in the coming years for sensitive zones. Zeolites in sidestream treatment can produce effluents with concentrations lower than 150 mg N-NH_4_/L, resulting in a direct reduction in energy consumption for biological removal processes.

Regarding regeneration, the concentration factor was higher in mainstream treatment than in the sidestream. However, since the feed concentration is higher in the sidestream, the final concentration of the regenerant solution is also higher, which favors subsequent valorization processes. In general terms, with a feed concentration of 50 mg N-NH_4_/L, a concentration of 1 g N-NH_4_/L can be achieved, whereas with a concentration of 800 mg N-NH_4_/L, it is possible to achieve concentrations of up to 4000 mg N-NH_4_/L.

Concerning the efficiency of recovery, it was possible to achieve recovery rates between 75% and 90% of the fed nitrogen. However, to understand the excess of nitrogen found in the regenerations in the sidestream it is necessary to analyze the accumulated N through the different cycles to achieve the final overall value of 80–84% N recovered.

To evaluate the quality of the regenerant solution for further treatment, both heavy metals (Table 8) and OMPs (Table S2) were analyzed.

As demonstrated in Table 8, heavy metals are not concentrated by the zeolites, and all of them remain below detection levels, except for aluminum. The presence of aluminum at 0.28 mg/L and 12.75 mg/L in the regenerant stream (from the mainstream and side stream, respectively) can be attributed to the partial dissolution of the zeolite caused by the high pH of the regenerant stream. In this case all the parameters comply with current legislation (considering thresholds stablished for sewage sludge and for fertilizers).

For organic micropollutants, the majority were detected below quantification levels in the regenerant for mainstream (Table S2) and sidestream (Table S4). However, the ion exchange process seemed to concentrate some micropollutants, including Celestolide and Ibuprofen. In contrast, other micropollutants like Sulfamethoxazole, Trimethoprim, and Citalopram were reduced to minimal levels, or even below the quantification threshold. The results for the sidestream can be found in the next section because they were characterized as the LLMC feed.

### 3.4. Zeolite Characterization

**Figure 5:**
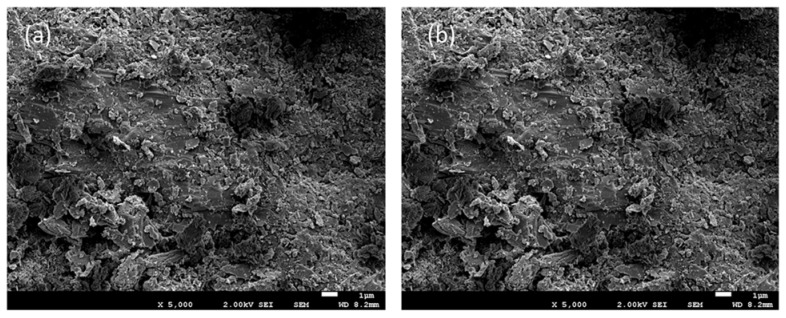

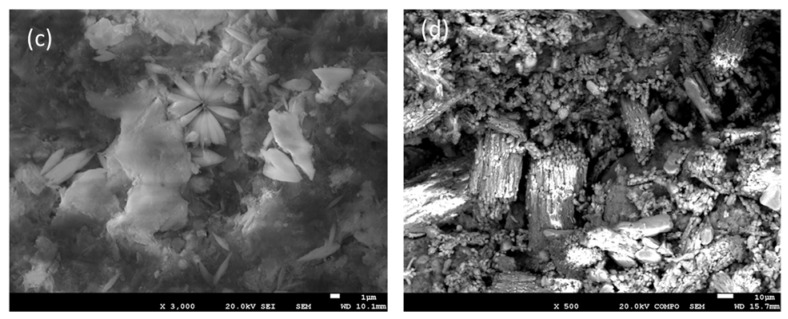
(**a**) New zeolite (×5000, 2 kV); (**b**–**d**) different pictures of zeolites after 4 cycles of operation prior to regeneration (×5000-2 kV, ×3000-20 kV, ×500-20 kV).

Zeolites have been extensively characterized, both in their pristine state and after undergoing experiments conducted in sidestream conditions. SEM results are reported in Figure 5.

As depicted in Figure 5, repeated operation cycles lead to the formation of numerous precipitates on the surface of the zeolites prior to regeneration. These precipitates primarily consist of CaCO_3_, which has a propensity to precipitate under alkaline conditions that occur during each NaOH-based regeneration process. This observation suggests that incorporating acid washing into the operational protocol after several cycles could be beneficial. Acid washing could help mitigate the formation of precipitates, thereby reducing the risk of bed compaction.

### 3.5. Liquid-Liquid Membrane Contactors Performance in Sidestream

In this section, the findings from the two fully characterized experiments conducted prior to comprehensive testing of the LLMCs are presented. These initial experiments aimed to validate the performance of the LLMCs and provide insights into the system’s capabilities and limitations, laying the groundwork for further optimization of the process.

The results from these preliminary experiments are discussed in detail, highlighting key observations and trends in ammonia removal efficiency. By examining these outcomes, the effectiveness of the LLMCs can be better understood and it is possible to identify areas for potential improvements in the pilot plant setting.

**Figure 6:**
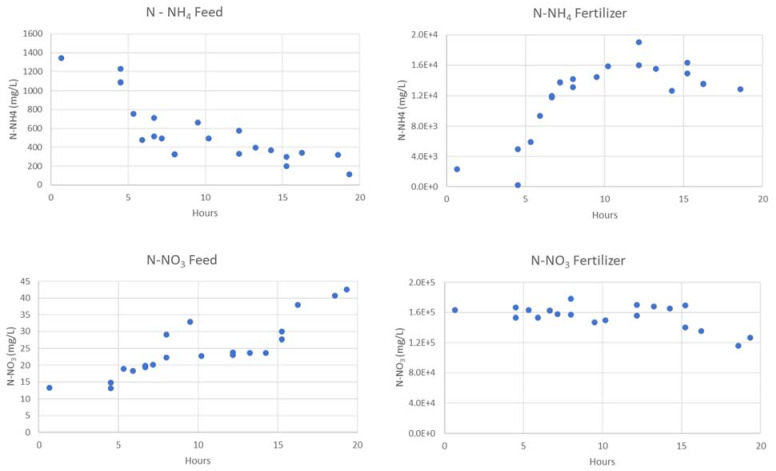
Evolution of N species in feed and fertiliser at 1.3 g N-NH_4_/L feed.

**Figure 7:**
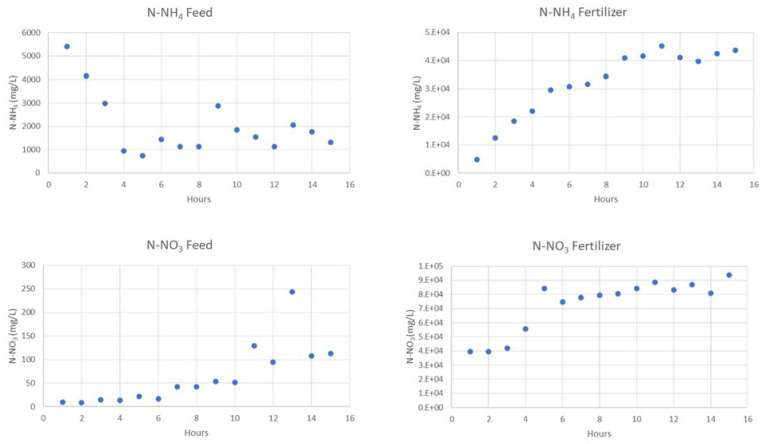
Evolution of N species in feed and fertilizer at 5.4 g N-NH_4_/L feed.

The evolution of N species in feed and fertiliser sides, for the first two experiments are presented in Figure 6 and Figure 7. Moreover, a complete characterization is displayed in Table 9 and Table 10.

All the experiments in this study were conducted using regenerant solution from zeolites at 4–5 g/L N-NH_4_ (sidestream). However, the first experiment had an initial concentration of only 1.3 g/L N-NH_4_, as the solution was stored at pH 12 for an extended period, resulting in nitrogen losses due to ammonia gas release. Despite this ammonia loss, the first experiment achieved a removal rate of 91.6%. In the second experiment, with an initial N-NH_4_ concentration of 5.4 g/L, the removal rate reached 75.6%. Higher initial concentrations generally result in lower removal rates.

**Figure 8:**
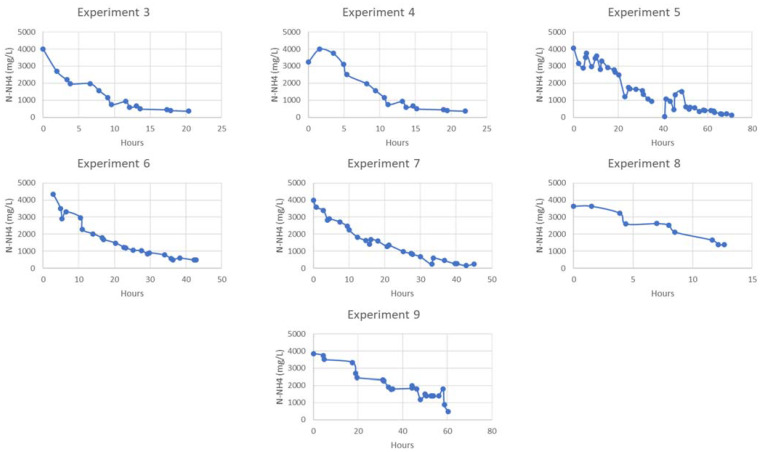
Nitrogen evolution in the rest of the experiments.

For experiments 3–9, the feed side performance was monitored using kits, with a full characterization of the final product conducted at the end. The evolution of N-NH_4_ for these experiments can be seen in Figure 8.

As shown in Table 11, ammonia removal reaches values of up to 96.6% of the ammonia in the feed after 70 h of operation. Table 12 presents the composition of the produced fertilizers in terms of ammonium, nitrate, and total nitrogen content. Lastly, Tables S3 and S4 provide an overview of the ionic composition as well as OMPs characterization of the produced fertilizers, respectively.

Regarding the membrane performance, the pilot plant was designed with the capability of developing chemical cleanings to the membrane. However, after a whole year of operation, there was no necessity of developing any chemical cleaning. This is due to membranes were fed with clean water (tap water) used for the regeneration of the zeolites thus there was a very low fouling potential. This fact is reinforced by the fact that membranes were not meant to filter the water but only to allow the gas passage which reduce the clogging of the membrane pores. To keep the good performance of the membrane it was identified that the most important is to carry out a drying step between experiments.

Vecino et al. [11] tested the same membranes with a similar initial concentration of 4 g N-NH_4_/L with both phosphoric and nitric, achieving 70–76% recovery with both acids in a single step and up to 93% recovery with phosphoric.

Boehler et al. [21] conducted a pilot plant study on the removal of ammoniacal nitrogen from wastewater treatment plant effluent using a membrane contactor unit. The study employed three hollow fiber membrane contactors in series, with a total surface area of 120 m^2^, and flow rates ranging from 5 to 12 L/m^2^h. To remove ammoniacal nitrogen in the form of ammonium sulfate, a sulfuric acid solution was passed through the lumen side. The pH of the wastewater was adjusted to around 9.5 to convert the ammonium species to ammonia gas, resulting in removal values of 95% or greater when the ammonium-N content was between 700 and 3400 mg/L. The study showed that the inclusion of a CO_2_ stripper section reduced the cost associated with addition of sodium hydroxide to elevate solution pH. However, the study also noted that precipitates formed in the stripper sections, which could lead to equipment fouling and clogging.

Norddahl et al. [37] also conducted a pilot plant study using a polypropylene hollow fiber membrane system to remove ammoniacal nitrogen from water. The study used water from either an anaerobic digester which was subsequently filtered by an ultrafiltration unit or a centrifuged sample from a sludge generated by a municipal solid waste treatment plant. The strip solution consisted of 1% (*w*/*w*) sulfuric acid. The study found that pH values of 10 or greater resulted in substantial acceleration of the removal of ammoniacal nitrogen due to the almost total formation of free ammonia.

Ulbricht et al. [38] summarized the results from a commercially operating membrane contactor system located in Wuppertal, Germany. The study employed two membrane contactors in series, which treated between 5 and 10 m^3^/h of water containing 500–2000 mg/L ammonia at a temperature of 40–50 °C and minimal levels of particulates. The feedwater was pH adjusted to 9 or greater, facilitating ammonia removal values of up to 95%. A common theme from the pilot plant studies was the need to raise the feed solution pH to at least 9, and preferably 10, to convert the majority of ammonium ion to ammonia gas.

To summarize, the pilot plant studies showed that membrane contactor technology is effective for the removal of ammoniacal nitrogen from wastewater treatment plant effluent and other sources of water. However, it requires the feed solution pH to be raised to at least 9, and preferably 10, to convert the ammonium species to ammonia gas. Additionally, the inclusion of a CO_2_ stripper section can reduce the cost associated with the addition of sodium hydroxide to elevate solution pH, but it can also lead to precipitate formation, which could cause equipment fouling and clogging.

The results demonstrated the potential to achieve high removal rates in the membranes, with values reaching up to 96%. It is evident that dosing acid to maximize mass transfer results in a final product with a high nitrogen content. This product can be directly used as a fertilizer or as a raw material for fertilizer production.

Upon examining the ionic characterization results, the impact of water transport becomes apparent. Each experiment began with 10 L of 0.4 M HNO_3_ in the acid side to initiate operation, after which the control loop added HNO_3_ (58%) to maintain a pH of 3.4. The dosed acid ranged from 12 to 15 L. Considering that each experiment produced 50 L of fertilizer, it can be concluded that each experiment experienced water transport of 20–25 L. As it has been reviewed in the literature research this is one of the main aspects to control the quality and the concentration of the fertilizer produced [39].

The overall composition of OMPs in the fertilizer remains undetectable despite water transport, ensuring its quality. This is supported by the presence of Ciprofloxacin and Oxytetracycline in the zeolites concentrate, but not in the fertilizer. Heavy metals appear in very low concentrations and are near the detection limit, while nutrient ions such as K^+^, Mg^2+^, SO_4_^2−^, and Ca^2+^ are present in certain concentrations. Sodium is the most significant ion, with concentrations of 3–4 g/L, attributed to the impact of water transport and high concentration of NaOH in the feed side. Spain’s fertilizers are regulated under Real Decreto 506/2013, which establishes maximum allowed limits for metals and other elements. When comparing the levels of metals in the provided table, it can be observed that aluminum, arsenic, cadmium, cobalt, chromium, copper, nickel, lead, and zinc in all fertilizers are below the maximum allowed limits.

Considering these findings, it would be worthwhile to explore the use of alternative acids, such as sulfuric acid, to evaluate whether water transport can be reduced. Additionally, adjusting the pH from 3 to 7 could potentially result in neutral solutions with the same molar concentrations of N-NH_4_ and N-NO_3_. Lastly, it would be interesting to consider lowering the NaOH concentration in the zeolites to reduce the sodium concentration in the LLMC feed solution, thus minimizing its passage with water. This approach could also decrease NaOH consumption and the NH_3_ losses associated with high pH.

## 4. Conclusions

This study has demonstrated the potential of using zeolites for ammonium recovery in both mainstream and sidestream processes of WWTPs. The adsorption efficiency and capacity of the zeolites were compared under different operating conditions, revealing that sidestream treatment yielded better results due to higher ammonium concentrations.

The pilot plant experiments showed that the CEC was significantly higher for sidestream treatment compared to mainstream treatment. With sidestream treatment, CEC values of around 20 mg N-NH_4_/g were achievable, while the lifespan of the zeolites was 4 cycles. Mainstream treatment, on the other hand, could produce effluents with less than 1 mg N-NH_4_/L, which is important considering the possibility of more stringent discharge limits in the future.

Regeneration efficiency was found to be between 75% and 80% of the fed nitrogen, with the concentration factor being higher in mainstream treatment. However, due to the higher feed concentration in sidestream treatment, the final concentration of its regenerant solution was also higher, favoring subsequent valorization processes.

The quality of the regenerant solution was assessed by analyzing heavy metals and organic micropollutants. Most heavy metals remained below detection levels, except for aluminum, which was attributed to partial dissolution of the zeolite due to the high pH of the regenerant stream. OMPs were generally detected below quantification levels, although some, like Celestolide and Ibuprofen, were concentrated during the ion exchange process.

Integration of zeolite regeneration with the LLMC system for fertilizer production appears to be a promising approach for wastewater treatment. The zeolite regeneration process effectively captures ammonia, producing a regenerant solution that can then be used as feed for the LLMC system to produce fertilizers.

The preliminary experiments on the LLMC system show removal rates of up to 96.6% of the ammonia in the feed after 70 h of operation. The produced fertilizers exhibit varied concentrations of ammonium, nitrate, and total nitrogen content, and show varying levels of heavy metals and other contaminants.

By combining the zeolite regeneration process with the LLMC system, an integrated approach for advanced wastewater treatment can be established. This approach can result in the production of valuable fertilizers, while simultaneously treating wastewater and reducing environmental pollution. Further research and optimization are needed to improve the efficiency and cost-effectiveness of this integrated system, but the initial results demonstrate its potential for application in wastewater treatment and resource recovery.

## Figures and Tables

**Table 1:** Sorption—desorption flows for each test.

Experiment	Sorption Flow (BV/h)	Regeneration Flow (BV/h)
4-2	4.1 ± 0.3	2.0 ± 0.2
4-0.8	3.9 ± 0.1	0.8 ± 0.1
10-5	10.1 ± 0.1	5.1 ± 0.3
10-2	9.9 ± 0.1	1.9 ± 0.2

**Table 2:** Zeolites experimental design.

	Mainstream	Sidestream
Adsorption—desorption cycles	4	4
Granulometry (mm)	1.0–2.5	0.5–1.0
Adsorption flow (L/h)	400	400
Regeneration flow (L/h)	700	700
Regenerant concentration (NaOH) (M)	0.5	0.2

**Table 3:** LLMCs experimental design.

	Experiment		
	1	2	3–9
N-NH_4_ feed concentration (g N-NH_4_/L)	1.3	4–5	4–5
Feed pH	12	12	12
Acid	HNO_3_	HNO_3_	HNO_3_
Feed flow (L/h)	500	500	500
Acid flow (L/h)	500	500	500
Acid side initial volume (L)	10	10	10
Feed side initial volume (L)	1000	1000	1000
Sampling points	Along the whole experiment	Along the whole experiment	Initial-final

**Table 4:** Sidestream water characterization.

Parameter	Mainstream	Centrates
Conductivity (mS/cm)	2.2	10.1
pH	8.4	8.6
Ca^2+^ (mg/L)	222	106.5
COD (mg/L)	66	777.3
K^+^ (mg/L)	28	545.8
Mg^2+^ (mg/L)	48	29.0
N-NH_4_ (mg N/L)	66	874.3
P-PO_4_ (mg P/L)	1	29.0
SO_4_^2−^ (mg/L)	151	168.4
TSS (mg/L)	30	800

**Table 5:** Recovery efficiency at lab-scale at different flow rates.

	Adsorption		Regeneration			
Experiment	Flow (BV/h)	CEC (mg/g_ze_)	Flow (BV/h)	N_recovered_ (mg/g_ze_)	Concentration Factor	Recovery (%)
4-2	4.1 ± 0.3	6.4 ± 1.1	2.0 ± 0.2	3.7 ± 0.2	4.8 ± 0.3	59.6 ± 12.7
4-0.8	3.9 ± 0.1	4.4 ± 1.3	0.8 ± 0.1	2.7 ± 0.9	3.4± 1.3	60.2 ± 17.6
10-5	10.1 ± 0.1	5.1 ± 0.2	5.1 ± 0.3	3.1 ± 0.4	2.4 ± 0.2	61.2 ± 10.2
10-2	9.9 ± 0.1	7.6 ± 0.2	1.9 ± 0.2	5.0 ± 0.9	5.3 ± 0.6	66.1 ± 13.8

**Table 6:** Results from zeolites in mainstream and sidestream.

**Mainstream**				
Cycles of operation	1	2	3	4
Inlet mean N-NH_4_ (mg/L)	58.9	66.5	57.9	55.9
CEC (g N-NH_4_/kg zeo)	5.3	5.3	4.5	3.2
N recovered (g N-NH_4_/kg zeo)	3.9	3.7	4.1	2.9
Regeneration level (%)	74	70	92	90
CF	15.7	16.3	19.8	13.8
**Sidestream**				
Cycles of operation	1	2	3	4
Inlet mean N-NH_4_ (g/L)	0.74	0.68	1.12	0.98
CEC (g N-NH_4_/kg zeo)	21.1	15.3	11.9	8.9
N recovered (g N-NH_4_/kg zeo)	8.2	19.4	10.25	10.4
Regeneration level (%)	38.8	126.4	86.1	117.5
CF	6.5	3.0	0.9	1.9

**Table 7:** Overall process efficiency.

	**Mainstream**				
Cycles	1	2	3	4	total
N fed (mg N-NH_4_)	526	526	446	324	1823
N recovered (mg N-NH_4_)	388	367	411	292	1459
Overall recovery (%)					80
	**Sidestream**				
Cycles	1	2	3	4	total
N fed (mg N-NH_4_)	2112	1535	1190	885	5722
N recovered (mg N-NH_4_)	820	1940	1025	1040	4825
Overall recovery (%)					84

**Table 8:** Heavy metal characterization.

	Mainstream			Sidestream		
	Influent ZE	Efluent ZE	Regenerant Stream	Influent ZE	Efluent ZE	Regenerant Stream
Al (mg/L)	<0.052	0.090	0.283	0.03	0.02	12.75
Cr (mg/L)	<0.005	<0.005	<0.005	0.05	0.05	<0.01
Ni (mg/L)	<0.055	<0.055	<0.055	0.05	0.05	0.02
Cu (mg/L)	<0.052	<0.052	<0.052	0.08	0.05	0.04
Zn (mg/L)	<0.052	<0.052	<0.052	0.03	0.03	0.03
Cd (mg/L)	<0.005	<0.005	<0.005	<0.01	<0.01	<0.01
Hg (mg/L)	<0.003	<0.003	<0.003	<0.003	<0.003	<0.003
Pb (mg/L)	<0.005	<0.005	<0.005	<0.01	<0.01	0.03

**Table 9:** First LLMC experiment complete characterization.

	Feed		Fertiliser
Parameter	Initial	Final	Final
N-NH_4_ (mg/L)	1341	113	11,464
NO_3_^−^ (mg/L)	58,541	188.4	132,155
N-NO_3_ (mg/L)	13.2	42.5	29.841
Al (mg/L)	6.82	5.93	0.58
As (mg/L)	0.02	0.02	<0.01
B (mg/L)	0.92	0.98	0.1
Ca (mg/L)	1.23	1.31	32.61
Cr (mg/L)	0.01	0.01	0.63
Cu (mg/L)	0.01	0.01	0.04
Fe (mg/L)	<0.01	<0.01	2.37
K^+^ (mg/L)	219.02	216.2	57.13
Li^+^ (mg/L)	0.03	0.03	0.02
Mg^2+^ (mg/L)	<0.1	<0.1	11.8
Mn (mg/L)	<0.01	<0.01	0.17
Mo (mg/L)	0.01	0.01	0.02
Na^+^ (mg/L)	7078.1	6142.5	1062
Ni (mg/L)	0.02	0.03	0.31
Pb (mg/L)	<0.01	0.01	0.02
P (mg/L)	2.8	2.84	0.72
Rb (mg/L)	0.16	0.17	0.04
Se (mg/L)	<0.01	0.01	0.1
Si (mg/L)	99.09	121.9	6.02
S (mg/L)	110.2	122	37.23
Sr (mg/L)	0.32	0.29	0.55
Zn (mg/L)	0.01	0.01	2.58
F^−^ (mg/L)	0.481	0.605	0.333
Cl^−^ (mg/L)	250.1	285.3	105.3
NO^2−^ (mg/L)	<0.1	<0.1	<0.1
Br^−^ (mg/L)	2.552	<0.05	<0.05
PO_4_^3−^ (mg/L)	8.27	8.37	2.13
SO_4_^2−^ (mg/L)	330.60	366.00	111.69

Be, Bi, Co, Cd, La, Sb, Se, Ti and V were below the limit of quantification (0.01 mg/L).

**Table 10:** Second LLMC experiment complete characterization.

	Feed		Fertiliser
Parameter	Initial	Final	Final
N-NH_4_ (mg/L)	5.41	1.32	43.67
N-NO_3_ (mg/L)	9	113	9811
Al (mg/L)	0.01	<0.01	0.49
B (mg/L)	0.34	0.37	0.12
Ca^2+^ (mg/L)	27.14	21.18	31.95
Cr (mg/L)	0.01	0.01	0.29
Cu (mg/L)	0.01	0.01	0.03
Fe (mg/L)	<0.01	<0.01	1.36
K^+^ (mg/L)	132.58	141.78	29.96
Li^+^ (mg/L)	0.07	0.08	0.01
Mg^2+^ (mg/L)	12.10	1.16	7.13
Mn (mg/L)	0.12	0.01	0.12
Mo (mg/L)	0.01	0.01	0.04
Na^+^ (mg/L)	4979.70	8190.60	996.80
Ni (mg/L)	0.01	0.01	0.19
Pb (mg/L)	<0.01	<0.01	<0.01
P (mg/L)	0.62	2.00	1.61
Rb (mg/L)	0.07	0.09	0.02
Si (mg/L)	11.23	8.41	3.23
S (mg/L)	150.60	157.80	45.54
Sr (mg/L)	1.12	0.78	0.37
Zn (mg/L)	0.06	0.01	4.00
F^−^ (mg/L)	0.20	<0.02	<0.02
NO_2_^−^ (mg/L)	<0.1	<0.1	<0.1
Br^−^ (mg/L)	<0.05	<0.05	<0.05
PO_4_^3−^ (mg/L)	1.81	5.89	4.76
SO_4_^2−^ (mg/L)	451.80	473.40	136.63

Be, Bi, Co, Cd, La, Sb, Se, Ti and V were below the limit of quantification (0.01 mg/L).

**Table 11:** Characterization for all the fertilizers produced along the experimental campaigns.

Experiment	Initial N (mg N-NH_4_/L)	Final N (mg N-NH_4_/L)	N Removed (%)	Operating Time (h)
1	1.34	113	91.6	20
2	5.41	1320	75.6	15
3	3.99	369	90.8	21
4	3.23	369	88.6	23
5	4.06	138	96.6	70
6	4.34	490	88.7	43
7	4.00	250	93.8	46
8	3.63	1380	62.0	13
9	3.84	470	87.8	60

**Table 12:** Nitrogen composition as N-NH_4_, N-NO_3_(g/L) and total N_t_ of each fertilizer produced.

Experiment	N-NH_4_ (g/L)	N-NO_3_(g/L)	Nt (%)
1	11.5	29.8	4.1
2	43.6	93.8	13.7
3	15.3	95.0	11.0
4	8.2	65.8	7.4
5	12.4	101.4	11.4
6	14.4	140.5	15.5
7	13.2	69.3	8.3
8	18.4	148.0	16.6
9	12.3	127.1	13.9

## Data Availability

Data available upon request.

## Supplementary Materials

The following supporting information can be downloaded at: https://www.mdpi.com/article/10.3390/membranes13060580/s1. Table S1: Operation parameters of the membrane contactors unit, Table S2: Organic micropollutant characterization in mainstream, Table S3: Ionic composition of each fertilizer, Table S4: Organic micropollutants (OMP) composition of each fertilizer.

## References

[1] World Population Prospects: The 2017 Revision. https://www.un.org/development/desa/publications/world-population-prospects-the-2017-revision.html..

[2] Charles H., Godfray J., Beddington J.R., Crute I.R., Haddad L., Lawrence D., Muir J.F., Pretty J., Robinson S., Thomas S.M. (2010). Food Security: The Challenge of Feeding 9 Billion People. Science.

[3] Puchongkawarin C., Gomez-Mont C., Stuckey D.C., Chachuat B. (2015). Optimization-Based Methodology for the Development of Wastewater Facilities for Energy and Nutrient Recovery. Chemosphere.

[4] Basosi R., Fierro A., Jez S. (2014). Mineral Nitrogen Fertilizers: Environmental Impact of Production and Use. Fertil. Compon. Uses Agric. Environ. Impacts.

[5] Mo W., Zhang Q. (2012). Can Municipal Wastewater Treatment Systems Be Carbon Neutral?. J. Environ. Manag..

[6] Huang J., Kankanamge N.R., Chow C., Welsh D.T., Li T., Teasdale P.R. (2018). Removing Ammonium from Water and Wastewater Using Cost-Effective Adsorbents: A Review. J. Environ. Sci..

[7] Mosa A., El-Ghamry A., Tolba M. (2020). Biochar-Supported Natural Zeolite Composite for Recovery and Reuse of Aqueous Phosphate and Humate: Batch Sorption–Desorption and Bioassay Investigations. Environ. Technol. Innov..

[8] Jiang N., Shang R., Heijman S.G.J., Rietveld L.C. (2018). High-Silica Zeolites for Adsorption of Organic Micro-Pollutants in Water Treatment: A Review. Water Res..

[9] Mazloomi F., Jalali M. (2016). Ammonium Removal from Aqueous Solutions by Natural Iranian Zeolite in the Presence of Organic Acids, Cations and Anions. J. Environ. Chem. Eng..

[10] Guo X., Zeng L., Jin X. (2013). Advanced Regeneration and Fixed-Bed Study of Ammonium and Potassium Removal from Anaerobic Digested Wastewater by Natural Zeolite. J. Environ. Sci..

[11] Vecino X., Reig M., Bhushan B., Gibert O., Valderrama C., Cortina J.L. (2019). Liquid Fertilizer Production by Ammonia Recovery from Treated Ammonia-Rich Regenerated Streams Using Liquid-Liquid Membrane Contactors. Chem. Eng. J..

[12] Thornton A., Pearce P., Parsons S.A. (2007). Ammonium Removal from Digested Sludge Liquors Using Ion Exchange. Water Res..

[13] Li M., Zhu X., Zhu F., Ren G., Cao G., Song L. (2011). Application of Modified Zeolite for Ammonium Removal from Drinking Water. Desalination.

[14] Vaneeckhaute C., Lebuf V., Michels E., Belia E., Vanrolleghem P.A., Tack F.M.G., Meers E. (2016). Nutrient Recovery from Digestate: Systematic Technology Review and Product Classification. Waste Biomass Valorization.

[15] Cooney E.L., Booker N.A., Shallcross D.C., Stevens G.W. (1999). Ammonia Removal from Wastewaters Using Natural Australian Zeolite. II. Pilot-Scale Study Using Continuous Packed Column Process. Sep. Sci. Technol..

[16] Koon J.H., Kaufman W.J. (1975). Ammonia Removal from Municipal Wastewaters by Ion Exchange.

[17] Drioli E., Criscuoli A., Curcio E. (2006). Membrane Contactors: Fundamentals, Applications and Potentialities.

[18] Darestani M., Haigh V., Couperthwaite S.J., Millar G.J., Nghiem L.D. (2017). Hollow Fibre Membrane Contactors for Ammonia Recovery: Current Status and Future Developments. J. Environ. Chem. Eng..

[19] Licon Bernal E.E., Maya C., Valderrama C., Cortina J.L. (2016). Valorization of Ammonia Concentrates from Treated Urban Wastewater Using Liquid–Liquid Membrane Contactors. Chem. Eng. J..

[20] Sancho I., Licon E., Valderrama C., de Arespacochaga N., López-Palau S., Cortina J.L. (2017). Recovery of Ammonia from Domestic Wastewater Effluents as Liquid Fertilizers by Integration of Natural Zeolites and Hollow Fibre Membrane Contactors. Sci. Total Environ..

[21] Boehler M.A., Heisele A., Seyfried A., Grömping M., Siegrist H. (2015). (NH_4_)_2_SO_4_ Recovery from Liquid Side Streams. Environ. Sci. Pollut. Res..

[22] Garcia-González M.C., Vanotti M.B. (2015). Recovery of Ammonia from Swine Manure Using Gas-Permeable Membranes: Effect of Waste Strength and PH. Waste Manag..

[23] Böhler M., Hernandez A., Gruber W., Fleiner J., Seyfried A. WP4-Nitrogen Management in Side Stream D 4.3: Operation and Optimization of Membrane Ammonia Stripping. http://powerstep.arctik.tech/files/d4-3-operation-and-optimization-of-membrane-ammonia-stripping.pdf.

[24] Water Environment (2005). APHA Standard Methods for the Examination of Water and Wastewater.

[25] Mayor A., Vecino X., Reig M., de Arespacochaga N., Valderrama C., Cortina J.L. (2020). Ammonium Valorization from Urban Wastewater as Liquid Fertilizers by Using Liquid–Liquid Membrane Contactors. Hollow Fiber Membr. Contactors.

[26] Reig M., Vecino X., Gibert O., Valderrama C., Cortina J.L. (2021). Study of the Operational Parameters in the Hollow Fibre Liquid-Liquid Membrane Contactors Process for Ammonia Valorisation as Liquid Fertiliser. Sep. Purif. Technol..

[27] Du Q., Liu S., Cao Z., Wang Y. (2005). Ammonia Removal from Aqueous Solution Using Natural Chinese Clinoptilolite. Separ. Purif. Technol..

[28] Beler-Baykal B., Oldenburg M., Sekoulov I. (2010). The Use of Ion Exchange in Ammonia Removal under Constant and Variable Loads. Environ. Technol..

[29] Baykal B.B., Guven D.A. (1997). Performance of Clinoptilolite Alone and in Combination with Sand Filters for the Removal of Ammonia Peaks from Domestic Wastewater. Water Sci. Technol..

[30] Liberti L., Boari G., Passino R. (1982). Advanced Wastewater Treatment by Ion Exchange. Effluent Water Treat J..

[31] Hlavay J., Vigh G.Y., Olaszi V., Inczédy J. (1982). Investigations on Natural Hungarian Zeolite for Ammonia Removal. Water Res..

[32] Semmens M.J., Porter P. (1979). Ammonium Removal by Ion Exchange: Using Biologically Restored Regenerant. J. Water Pollut. Control Fed..

[33] Hedström A. (2001). Ion Exchange of Ammonium in Zeolites: A Literature Review. J. Environ. Eng..

[34] Wang S., Peng Y. (2010). Natural Zeolites as Effective Adsorbents in Water and Wastewater Treatment. Chem. Eng. J..

[35] Lei X., Li M., Zhang Z., Feng C., Bai W., Sugiura N. (2009). Electrochemical Regeneration of Zeolites and the Removal of Ammonia. J. Hazard. Mater..

[36] Huang H., Yang L., Xue Q., Liu J., Hou L., Ding L. (2015). Removal of Ammonium from Swine Wastewater by Zeolite Combined with Chlorination for Regeneration. J. Environ. Manag..

[37] Norddahl B., Horn V.G., Larsson M., Preez J.H., Christensen K. (2006). A Membrane Contactor for Ammonia Stripping. Pilot Scale Exp. Model..

[38] Ulbricht M., Schneider J., Stasiak M., Sengupta A. (2013). Ammonia Recovery from Industrial Wastewater by TransMembranechemiSorption. Chem. Ing. Tech..

[39] Guida S., Van Peteghem L., Luqmani B., Sakarika M., McLeod A., McAdam E.J., Jefferson B., Rabaey K., Soares A. (2022). Ammonia Recovery from Brines Originating from a Municipal Wastewater Ion Exchange Process and Valorization of Recovered Nitrogen into Microbial Protein. Chem. Eng. J..

