# Supplementary material for: Advanced Hybrid System for Ammonium Valorization as Liquid Fertilizer from Treated Urban Wastewaters: Validation of Natural Zeolites Pretreatment and Liquid-Liquid Membrane Contactors at Pilot Plant Scale
Source: Membranes (Basel). 2023 Jun 2;13(6):580. doi: 10.3390/membranes13060580 (PMC10304151; doi:10.3390/membranes13060580)
Supplement: Supplementary file 1 [file membranes-13-00580-s001.zip › membranes-2413387-supplementary.pdf]

Article

Advanced Hybrid System for Ammonium Valorization as Liquid Fertilizer from Treated Urban Wastewaters: Validation of Natural Zeolites Pretreatment and Liquid-Liquid Membrane Contactors at Pilot Plant Scale

Álvaro Mayor [1], Mònica Reig [2,3,*], Xanel Vecino [4], José Luis Cortina [1,2,3] and César Valderrama [2,3]

[1] CETaqua, Carretera d'Esplugues, 75, 08940 Cornellà de Llobregat, Spain; amayor@cetaqua.com (A.M.); jose.luis.cortina@upc.edu (J.L.C.)
[2] Chemical Engineering Department, Escola d'Enginyeria de Barcelona Est (EEBE) Universitat Politècnica de Catalunya (UPC)-BarcelonaTECH, C/Eduard Maristany 10-14, Campus Diagonal-Besòs, 08930 Barcelona, Spain; cesar.alberto.valderrama@upc.edu
[3] Barcelona Research Center for Multiscale Science and Engineering, Campus Diagonal-Besòs, 08930 Barcelona, Spain
[4] CINTECX, Chemical Engineering Department, Campus as Lagoas-Marcosende, University of Vigo, 36310 Vigo, Spain; xanel.vecino@uvigo.es
* Correspondence: monica.reig@upc.edu

1. Supplementary materials

Table S1 Operation parameters of the membrane contactors unit

Parameter	Value	Units
Feed/ acid volume ratio storage	100	L/L
Feed and acid flowrates	0.5 – 2	m^3/h
Temperature	18 – 25	ºC
Feed pH	>11	
Operating voltage	380	VAC-50 Hz-3F+N
Power	27.7	kW

Table S2 Organic micropollutant characterization in mainstream

OMP (µg/L)	Zeolite influent	Zeolite concentrate
Celestolide	<0.01	0.52
Galaxolide	0.16	<0.01
Tonalide	0.07	<0.01
Carbamazepine	0.15	0.16
Diazepam	0.01	<0.001
Ibuprofen	0.06	0.34
Naproxen	<0.005	<0.005
Diclofenac	2.83	<0.02
4-octylphenol	<0.004	<0.004
4-nonylphenol	<0.004	<0.004
Bisphenol A	<0.005	<0.005
Triclosan	<0.01	<0.01

Erythromycin	0.04	<0.001
Fluoxetine	0.03	0.01
Roxithromycin	<0.004	0.005
Sulfamethoxazole	0.14	0.04
Trimethoprim	0.27	0.07
Citalopram	0.19	<0.003
Estrona	<0.003	<0.002
Estradiol	<0.003	<0.002
ethinyl estradiol	<0.003	<0.002

Table S3 Ionic composition of each fertilizer

Compound (mg/L)	Fert 1	Fert 2	Fert 3	Fert 4	Fert 5	Fert 6	Fert 7	Fert 8	Fert 9
Al	0.02	<0.01	1.97	1.67	0.45	0.18	<0.01	0.66	1.96
As	0.02	0.01	0.02	0.02	0.01	0.02	0.02	0.03	0.02
B	0.05	<0.01	0.02	0.08	0.05	0.04	<0.01	0.03	0.05
Ca^{2+}	9.79	10.71	8.16	9.75	9.92	24.83	16.61	22.37	29.76
Cr	<0.01	<0.01	0.09	0.05	0.08	0.15	0.01	0.08	0.05
Cu	0.04	0.1	0.09	0.18	0.29	4.13	0.07	0.26	0.79
Fe	0.05	0.01	1.92	0.3	0.44	3.72	0.05	0.4	0.54
K^{+}	51.4	41.4	60.7	67.6	47.7	44.2	46.6	45.00	73.00
Li	0.01	0.01	0.01	0.02	0.02	0.02	0.02	0.02	0.02
Mg^{2+}	2.09	2.59	2.84	2.93	2.72	7.79	3.57	7.85	6.72
Mn	0.04	0.05	0.04	0.04	0.04	0.15	0.04	0.1	0.1
Mo	0.02	0.09	0.05	0.02	0.02	0.04	0.04	0.01	0.07
Na^{+}	3081	2462	3326	3940	3592	3224	3488	3391	4468
Ni	0.03	0.06	0.03	0.03	0.05	0.17	0.02	0.06	0.04
Pb	<0.01	<0.01	0.01	0.05	0.08	0.14	0.01	0.05	0.1
P	0.27	0.16	1.12	2.01	2.14	6.17	2.22	2.27	7.34
Rb	0.04	0.03	0.04	0.05	0.03	0.03	0.03	0.08	0.14
Si	10.93	6.46	13.13	24.3	27.66	18.66	17.76	12.16	30.89
S	35.21	19.54	25.24	39.79	40.83	40.12	31.71	32.86	33.68
Sr	0.16	0.15	0.11	0.16	0.13	0.42	0.32	0.4	0.58
Tl	0.01	0.01	<0.01	<0.01	0.01	0.01	0.02	0.01	0.01
Zn	0.18	0.4	0.15	0.54	0.85	3.41	0.11	0.95	1.86

Be, Bi, Co, Cd, La, Sb, Se, Ti and, V were below the limit of quantification (0.01 mg/L)

Table S4 Organic micropollutants (OMP) composition of each fertilizer

OMP (ng/L)	ZC*	Fert 1	Fert 2	Fert 3	Fert 4	Fert 5	Fert 6	Fert 7	Fert 8	Fert 9
Ciprofloxacin	705	44	130	21	34	20	16	46	<1.8	<1.8
Sulfadiazine	4	<0.5	<0.5	<0.5	<0.5	<0.5	<0.5	<0.5	<0.5	5
Oxytetracycline	697	<2.0	<2.0	<2.0	<2.0	<2.0	<2.0	<2.0	<2.0	<2.0
Sulfamethoxazole	<4.4	<4.4	<4.4	<4.4	<4.4	<4.4	<4.4	<4.4	<4.4	<4.4
Azithromycin	146	<4.0	<4.0	<4.0	<4.0	<4.0	<4.0	<4.0	<4.0	<4.0
Trimethoprim	23	<1.4	<1.4	<1.4	<1.4	<1.4	<1.4	<1.4	<1.4	<1.4
Citalopram	<1.8	9	10	<1.8	<1.8	<1.8	<1.8	11	9	14

Fluoxetine	<35.4	<35.4	<35.4	<10.6	<10.6	<10.6	<10.6	<35.4	<35.4	<35.4
Diazepam	<5.9	<5.9	<5.9	<5.9	<5.9	<5.9	<5.9	<5.9	<5.9	<5.9
Carbamazepine	<6.4	15	<6.4	<6.4	303	<6.4	<6.4	<6.4	<6.4	<6.4
Methiocarb	<11.1	<11.1	<11.1	<11.1	14	<11.1	<11.1	<11.1	<11.1	<11.1

*ZC: zeolite concentrate after regeneratio with NaOH
